# Microorganisms Resistant to Antimicrobials in Wild Canarian Egyptian Vultures (*Neophron percnopterus majorensis*)

**DOI:** 10.3390/ani10060970

**Published:** 2020-06-03

**Authors:** Alejandro Suárez-Pérez, Juan Alberto Corbera, Margarita González-Martín, José Antonio Donázar, Rubén Sebastián Rosales, Manuel Morales, María Teresa Tejedor-Junco

**Affiliations:** 1 Wildlife Animal Rescue Center, Cabildo de Gran Canaria, 35017 Las Palmas de Gran Canaria, Canary Islands, Spain; alejandro.suarezperez@ulpgc.es; 2 Department of Animal Pathology, Animal Production and Food Hygiene and Technology, University of Las Palmas de Gran Canaria (ULPGC), 35413 Arucas, Spain; ruben.rosales@ulpgc.es (R.S.R.); manuel.morales@ulpgc.es (M.M.); 3 Research Institute of Biomedical and Health Sciences, ULPGC, 35016 Las Palmas de Gran Canaris, Spain; margaritarosa.gonzalez@ulpgc.es (M.G.-M.); mariateresa.tejedor@ulpgc.es (M.T.T.-J.); 4 Department of Clinical Sciences, ULPGC, 35016 Las Palmas de Gran Canaris, Spain; 5 Department of Biological Conservation, Doñana Biological Station, Spanish Council for Scientific Research (CSIC), 41092 Seville, Spain; donazar@ebd.csic.es

**Keywords:** antimicrobials, antibiotic, resistance, *Escherichia coli*, *Salmonella*, Canarian Egyptian vulture, *Neophron percnopterus majorensis*, vulture, guirre

## Abstract

**Simple Summary:**

Antibiotics are used to prevent and treat bacterial infections in both animals and humans. If bacteria become resistant to them, in particular as the result of the misuse and overuse of antibiotics, the infections that they cause are harder to treat. Therefore, the detection of microorganisms resistant to antimicrobial drugs is an important issue, considering the interaction among domestic animals, human, the ecosystem and wild animals. Wild birds, and particularly birds-of-prey, are sentinels, reservoirs, spreaders and a source of infection for human beings and other animals. Wildlife can also act as an asymptomatic reservoir for zoonotic bacteria (e.g., *Salmonella*). The presence of antimicrobial-resistant microorganisms was investigated for a period of three years and the differences between chicks in the nest (*n* = 81) and adult and immature birds (*n* = 61) were analyzed. Gram negative bacteria were isolated in all the samples. *Escherichia coli* was obtained in 80.28% of the samples, where the prevalence of *Salmonella* in our study was 6.3%. The results of our study support the idea that raptors could act as reservoirs of *Salmonella* and antimicrobial-resistant bacteria, posing a risk not only to wildlife but also to livestock and the human population.

**Abstract:**

Due to their predatory habits, raptors may serve as indicators of the presence of antimicrobial-resistant bacteria in the environment, but they also represent a public health risk for livestock and humans because they can act as reservoirs, sources and spreaders of these bacteria. Our objective was to determine the presence of antimicrobial-resistant bacteria in cloacal samples of Canarian Egyptian vultures (*Neophron percnopterus majorensis)*, an endemic bird of prey. One hundred and forty-two cloacal swabs were obtained; *Escherichia coli* was isolated from 80.28% and *Salmonella* from 6.3% of these samples. Low levels of susceptibility to ampicillin, tetracycline and trimethoprim/sulfamethoxazole were found. About 20% of the isolates were resistant or presented intermediate susceptibility to fluoroquinolones. Surprisingly, we found isolates resistant to imipenem (6.96%). Isolates from chicks were more susceptible to antimicrobial drugs than adult and immature birds. About 50% of *E. coli* isolates were resistant to ampicillin, tetracycline and trimethoprim/sulfamethoxazole, and about 20% to piperacillin, enrofloxacin and marbofloxacin. High percentages of isolates of *Salmonella* were found to be resistant to cephalexin (88%) and aminoglycosides (greater than 77%). Our results support the idea that raptors could act as reservoirs of *Salmonella* and antimicrobial-resistant bacteria, posing a risk not only to wildlife but also to livestock and the human population, thus reinforcing the need to minimize the exposure of wildlife to antimicrobial agent through human and livestock waste.

## 1. Introduction

The Canarian Egyptian vulture (*Neophron percnopterus majorensis*) is an endemic bird-of-prey in the Canary Islands, known locally as the “guirre”. Based on its morphological and genetic characteristics, this vulture is a subspecies of the Egyptian vulture (*Neophron percnopterus*) [1]. It is a necrophagous bird that feeds to a large extent on the remains of dead animals, assiduously visiting farms and supplementary feeding stations [2]. In addition, it is known to hunt small prey and feed on animal excrements. This source of anthropogenic food has a direct influence on the diet, behavior, distribution, density and health of the population, thus resulting in variability in terms of their demographics and population dynamics [2,3]. The *Neophron percnopterus majorensis* (“guirre” or “alimoche canario”) is included under the category of “In Danger of Extinction” in the Spanish Catalogue of Endangered Species [4]. *Neophron percnopterus* is included under the category of “Endangered” in the International Union for Conservation of Nature’s (IUCN) Red List of Threatened Species.

Antimicrobial resistance is a “One Health” issue [5]. “One Health” is an emerging concept that sustains that the human, animal and environmental health are all interconnected, and that a global strategy is needed to face this challenge [6]. Even when it is accepted that the most important factor triggering the appearance and spread of antimicrobial-resistant bacteria in human medicine is the use of antibiotic therapies in human patients, we have to consider that the antimicrobials used in Veterinary Medicine contribute to worsening the problem [7]. Therefore, the detection of microorganisms resistant to antimicrobials is important, because of the interaction among domestic animals, human, the ecosystem and the wild animals [8]. Moreover, the “One Health” approach and the role of biodiversity in promoting the human health ought to be examined and requires further research [9].

**Figure 1:**
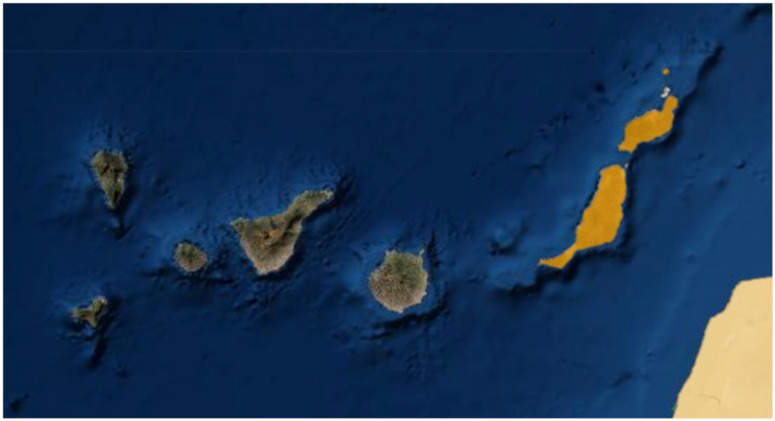
Canarian Egyptian vulture inhabits only two islands (Fuerteventura and Lanzarote) of the Canary Island Archipelago. (From: Leaflet|Powered by Esri|Esri, HERE, Garmin, FAO, NOAA, USGS).

Regarding to antimicrobial resistance, wild birds can be important as sentinels, reservoirs, spreaders and sources of infection for human beings and other animals [10,11]. The migratory behavior of raptors has been proposed as a mechanism for disseminating antimicrobial-resistant bacteria [8]. Our study is focused on the Canarian Egyptian vulture, which is not migratory and inhabits two islands (Fuerteventura and Lanzarote) of the Canary Island Archipelago (Figure 1).

Microorganisms present in the vulture may exhibit resistance to antibiotics, which could constitute a threat to global health, food security and development, as defined by the WHO [12]. Over the last decade, an increase in extended-spectrum β-lactamases (ESBL) and pAmpC producing *Escherichia coli* has been detected in humans, pets, food-producing animals and derived meat products [13,14]. In Spain, a high percentage of birds are carriers of ESBL or pAmpC *Escherichia coli*, especially in the case of storks (*Ciconia* spp.), kites (*Milvus* spp.) and vultures. This research showed that birds could contribute to the global spread of ESBL/pAmpC producing *E. coli* in natural ecosystems [15].

Wildlife can act as an asymptomatic reservoir for zoonotic bacteria, such as *Salmonella,* and this bacterium can also be a cause of disease [16]. A study conducted at a Wildlife Rehabilitation Center [16], showed a prevalence of 33.3% of this bacterium in the Griffon Vulture (*Gyps fulvus*), as well as a high percentage of multiresistant *Salmonella* isolates. Vidal et al. [17] found that *E. coli* was the most common microorganism recovered from necropsies of raptors with clinical signs of septicemia or respiratory disorders. In this research, 50% of all the isolates, and 71% of *E. coli*, showed multidrug resistance. Fecal contamination of the environment by wild birds could play a role in the transmission of pathogens to humans, via livestock or water supplies. Some important mechanisms of resistance are encoded by genes located on mobile elements and this allows the transference of resistance genes from commensal or opportunistic bacteria like *E. coli* to pathogenic ones [18]. Investigations on the prevalence and drug resistance of *Salmonella* in birds-of-prey could help us to understand the role of birds-of-prey in its epidemiology in terms of the wildlife–human interface [19].

Information on antimicrobial resistance pertaining to Gram-negative bacteria from wild raptors has been obtained mainly from birds at rescue centers, but only a few studies have investigated this topic in raptors living in their natural ecosystems. This knowledge is needed to assess the importance of wild raptors as reservoirs and transmitters of antimicrobial-resistant bacteria, to determine their potential impact on public health and their role as a potential health threat to workers involved in the protection of this animal species [20]. Therefore, the main objective of this study was to determine the presence of antimicrobial-resistant bacteria in cloacal samples of Canarian Egyptian vultures, with a special focus on *E. coli* and *Salmonella*. We also compared the resistance throughout the three years of the study and analyzed the differences among chicks in the nest and adult or immature birds.

## 2. Materials and Methods

Cloacal samples of vultures were collected during the years 2015 (30 samples), 2016 (62 samples) and 2017 (50 samples), as part of a long-term monitoring program of this population [21].

The birds were classified by age as chicks, immature birds—which include juveniles (less than 2 years of age) and sub-adults (between 2 and 5 years of age)—and adults (over 5 years of age). The age of the animals was determined according to the plumage characteristics and the color of the beak and the eye [22]. Adult and immature birds (juveniles and sub-adults) were trapped in baited locations by means of cannon-netting. Chicks were captured during the fledgling stage in the nests. All the birds captured were apparently healthy individuals. All procedures, including the capture and handling methods for wild vultures, were carried out under the Project License approved by The Biodiversity Directorate of the Government of the Canary Islands; the official reference numbers from the competent authority are: 2014/256, 2015/1652 and 2016/1707.

A total of 142 cloacal swabs were obtained, of which 81 were from chicks on the nests and 61 were from wild adult or immature birds. Cloacal swabs were placed in Amies blue plastic/viscose gel transport medium (reference number 50 710-0432, VWR^TM^, Darmstadt, Germany)) and conserved at 4 °C until they reached to the Microbiology Laboratory (within 24 h).

Samples were cultured on MacConkey agar and Hektoen agar and incubated overnight at 37 °C. Rapapport Broth (bioMérieux, Marcy L’Etoile, France) was also inoculated and incubated overnight at 42 °C, and was later streaked onto Hektoen agar (BD Difco, Detroit, MI, USA).

Bacteria were identified using the Vitek^®^2 automated system (bioMérieux, Marcy L’Etoile, France), with VITEK^®^ 2 GN ID Cards, following the manufacturer’s instructions. These cards allow for the identification of more than 150 species of clinically relevant Gram-negative bacilli.

Antimicrobial susceptibility tests were performed using the disk diffusion method in accordance with the Clinical and Laboratory Standards Institute (CLSI 2015) guidelines [23]. The inoculum was prepared in saline solution, using isolated fresh colonies and with the turbidity adjusted to a 0.5 McFarland standard. Using a sterile swab, the surface of an Müller–Hinton agar plate (BD Difco, Detroit, MI, USA) was streaked with the inoculum. Later, the antimicrobial-impregnated disks were applied and the plates were incubated in a stove at 37 °C for 24 h. The inhibition zones were measured and scored as susceptible, intermediate or resistant, according to the guidelines established by the CLSI.

The following 16 antibiotics were tested: ampicillin (10 µg), amoxicillin/clavulanic acid (20 µg + 10 µg), cephalexin (30 µg), cefpodoxime (10 µg), piperacillin (100 µg), imipenem (10 µg), gentamicin (10 µg), tobramycin (10 µg), amikacin (30 µg), enrofloxacin (5 µg), marbofloxacin (5 µg), tetracycline (30 µg), nitrofurantoin (300 µg), chloramphenicol (30 µg), polymyxin B (300 U) and trimethoprim/sulfamethoxazole (23.75 µg + 1.25 µg).

For the comparison of the results of the antimicrobial resistance related to the age of the animals, the chi-square with the Yates’s correction for continuity was calculated. SPSS v.22.0 (SPSS Inc., Chicago, IL, USA) was used to compare datasets. Statistical significance was set at *p* < 0.05. Additionally, statistical differences in the antimicrobial resistances of *Salmonella* compared with the rest of the isolated species were studied.

## 3. Results

The results of this study identify antimicrobial resistance in Gram-negative bacteria isolated from cloacal samples of vultures on the Canary Island Archipelago (Spain).

Gram-negative bacteria were isolated in all the samples. Biochemical identification was carried out for one colony per plate. One hundred and fourteen (80.28%) isolates were identified as *E. coli*, and nine isolates (6.3%) were determined to be *Salmonella* spp., while the remaining 19 isolates (13.42%) included *Proteus mirabilis, Providencia rettgeri, P. stuartii, Klebsiella pneumoniae, K. oxytoca, Raoultella ornithinolytica* and *Citrobacter braakii.*

**Figure 2:**
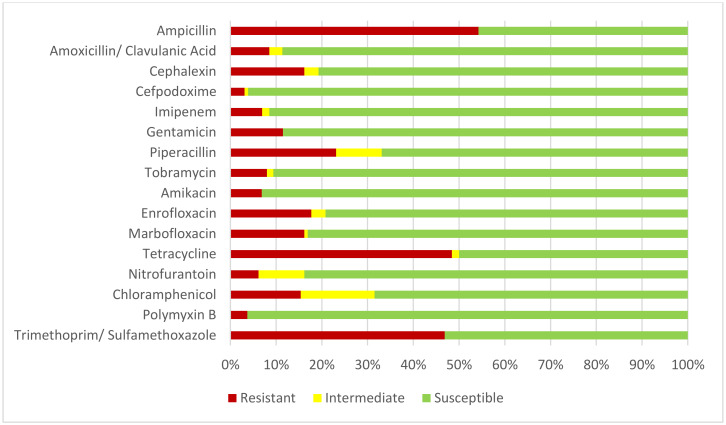
Global results of susceptibility tests. Antimicrobial susceptibility (%) of all isolates tested.

In order to present our results from different perspectives, the percentages of resistant bacteria were analyzed in relation to the global susceptibility (Table 1), the year of the study (Table 2), the age of the animals (Table 3) and the different bacterial species (Table 4). The highest antimicrobial resistance percentages in the isolates were observed for ampicillin (54.25%), tetracycline (48.44%) and trimethoprim/sulfamethoxazole (46.85), whereas the lowest antimicrobial resistance percentages corresponded to cefpodoxime (3.1%), polymyxin B (3.7%) and nitrofurantoin (6.15%). The results of all the antimicrobials studied are shown in Table 1 and Figure 2.

**Figure 3:**
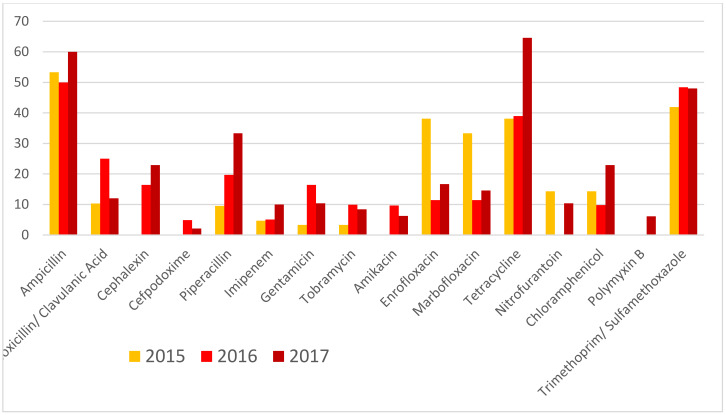
Variations in the percentages of resistant during the three years of the study.

Variations in susceptibility percentages during the three years of the study are shown in Table 2 and Figure 3. From 2015 to 2017, a sharp decrease in susceptibility was found for cephalexin, piperacillin, tetracycline, nitrofurantoin and chloramphenicol. On the other hand, an increase in the percentages of susceptibility to enrofloxacin and marbofloxacin was observed.

**Figure 4:**
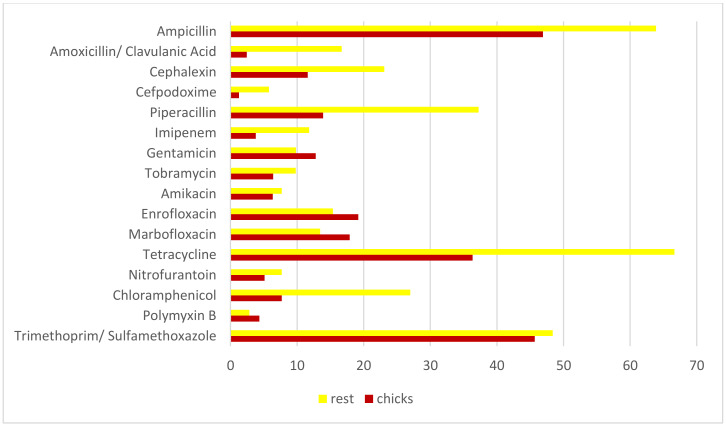
Differences in the percentage of resistant between chicks in the nests and the rest of the of animals (adult and immature birds).

Table 3 and Figure 4 shows the susceptibility of bacteria isolated from chicks in the nests and from the rest of animals (adult and immature birds). Isolates from chicks were more susceptible to ampicillin, amoxicillin/clavulanic acid, cephalexin, piperacillin, tetracycline, nitrofurantoin and chloramphenicol than those from the rest of animals. However, significant statistical differences were only found in amoxicillin/clavulanic acid, piperacillin, tetracycline and chloramphenicol.

**Figure 5:**
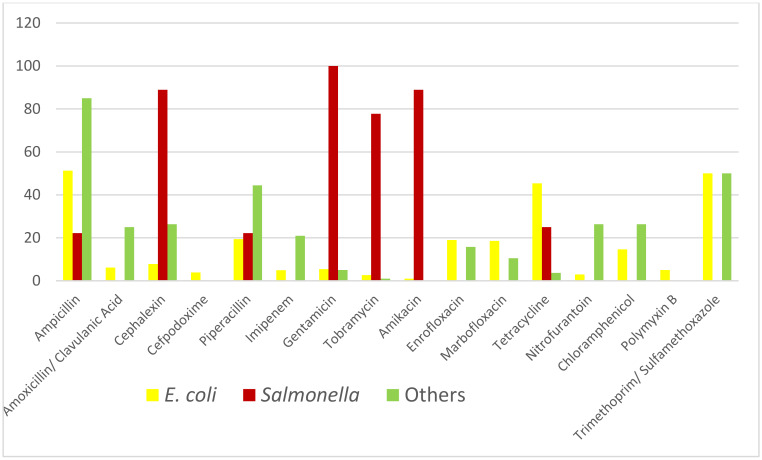
Differences in percentages of resistant between bacteria species.

Lastly, the differences in susceptibility found among *E. coli*, *Salmonella* and the rest of species are summarized in Table 4 and Figure 5. About 50% of *E. coli* isolates were resistant to ampicillin, tetracycline and trimethoprim/sulfamethoxazole and about 20% were resistant to piperacillin, enrofloxacin and marbofloxacin. High percentages of isolates of *Salmonella* resistant to cephalexin and aminoglycosides were also found. We did not find any statistically significant differences in the presence of *Salmonella* between animals of different ages (χ^2^ = 0.065; *p*-value = 0.799).

## 4. Discussion

The increase in multidrug-resistant bacteria in wildlife is a global threat, because it can lead to increased morbidity and mortality, and can also lead to increase healthcare costs [24]. In order to increase the available information on antibiotic resistance in wild animals, we have investigated this topic in isolates from fecal samples of Canarian vultures. With regard to the overall results of antimicrobial susceptibility tests (detailed in Table 1), low levels of susceptibility to ampicillin (45.75%), tetracycline (50%) and trimethoprim/sulfamethoxazole (53.15%) were found. Percentages of isolates susceptible to piperacillin and chloramphenicol were also low (66.9% and 68.47% respectively).

About 20% of isolates were resistant or presented intermediate susceptibility to fluoroquinolones (enrofloxacin and marbofloxacin). The use of veterinary antimicrobials is clearly regulated and withdrawal periods or residue levels are established only for animal by-products intended for human consumption. However, carcasses and other remains, frequently from diseased animals, can be consumed by raptors, which implies the ingestion of antimicrobial residues or resistant bacteria. Blanco et al. [25] evaluated the presence of fluoroquinolones in the plasma of nestling vultures feeding on domestic livestock carcasses and found residues of these pharmaceuticals in 92% of the animals sampled. Besides having to secondary or side effects, such as the alteration of microbiota, cartilage disorders, etc. [26], these compounds could select quinolone-resistant bacteria in vulture populations. We found a relatively high percentage of quinolone-resistant bacteria, which could be due to either direct contamination or the ingestion of quinolone-contaminated feed followed by the selection of quinolone-resistant bacteria in the vultures’ intestine.

Surprisingly, we also found isolates resistant to imipenem, an antibiotic restricted exclusively to hospital use. High levels of resistance to carbapenems by bacteria isolated from migratory birds have been described by other authors [27,28]. Imipenem resistance was low (6.9%) and found mainly in *E. coli*, *Proteus* and *Providencia*. As pointed out by Wang et al. [8], even though when resistance to carbapenems is still rare in wild animals, its emergence in wild birds is a matter of concern. The emergence and dissemination of carbapenemase-producing multidrug-resistant bacteria represent an important public health risk, causing serious infectious with very limited therapeutic options [29].

Dolejska et al. [30] described the isolation of 120 carbapenemase-producing *Enterobacterales* from silver gulls (*Chroicocephalus novae-hollandiae)* in Australia (120 isolates from 504 samples, 85 *E. coli*). The *bla*IMP-4 gene was found in 116 isolates, and it was also the most commonly detected gene among carbapenemase-producing *Enterobacterales* isolates in humans in that country. As described below, this high prevalence is probably due to the feeding habits of gulls. Prevalence in Canarian vultures was low, probably because they usually feed on the remains of dead animals, but it is possible that in some cases they might eat rubbish and acquire carbapenem-resistant bacteria. Since vultures can also hunt small prey, it would also be possible for the prey to harbor imipenem-resistant bacteria, due to their close contact with human habitats. Therefore, the feeding habits of gulls, which usually forage in rubbish dumps, could explain this difference.

Resistance to amikacin, which has been withdrawn from community use, is also relevant. It has been demonstrated than the use of aminoglycosides in human and veterinary medicine is associated with an increase in resistance to this group of antimicrobials [31]. Aminoglycosides, such as amikacin or gentamicin, are important antimicrobials for the treatment of human and animal infections, and are classified by the WHO as critically important antimicrobials for human medicine [32].

When we focused on the variations in susceptibility percentages (Table 2), there was a decrease in susceptibility for cephalexin, piperacillin, tetracycline, nitrofurantoin and chloramphenicol, as well as increased susceptibility to enrofloxacin and marbofloxacin. These results could be a consequence of the recommendations of the European Parliament on the prudent use of veterinary antibiotics, which were published in 2019, but discussed throughout the previous years (Regulation (UE) 2019/6) [33].

In our study, the bacteria isolated from chicks were more susceptible to several antibiotics (Table 3) than those from the rest of animals. While, Marrow et al. [34] found no differences in the frequencies of antimicrobial-resistance between different age groups when studying *Enterococcus* in raptors, differences has been observed in the transmission of different antimicrobial-resistant enteric bacteria from cattle and the environment to wild birds, in particular between *E. coli* and *Enterococcus* [35]. To our knowledge, no further information is available on the differences in resistance among isolates from raptors of different age groups. We suggest that the differences found could be due to different feeding habits. Although the origin of bacterial resistance genes in wild animals is still unclear, if animals have not been treated with antibiotics directly, direct ingestion of resistant bacteria through contaminated food, ingestion of antimicrobial compounds or contact with sewage or animal manure might contribute to this problem. However, chicks in the nests have less contact with livestock manure or sewage. Moreover, since they are younger, they will have been eating foods that contain antibiotics for a shorter period of time. These facts could explain to some extent the differences in susceptibility between bacteria from chicks and the remaining animals found in our study. Further studies on the time and level of antibiotic exposure required to generate antibiotic resistance are needed.

With regard to the prevalence of different bacterial species, *E. coli* was obtained in 80.28% of the samples. This high prevalence is in agreement with the results of Sharma et al. [36] for Egyptian vultures (*Neophron percnopterus*) in India and the results found by Radhouani et al. [18] in samples taken from common buzzards (*Buteo buteo*) in Portugal. However, other authors describe a lower prevalence of *E. coli*, and it seems that the differences in prevalence do not depend on whether the samples come from birds of prey from countries with different human and livestock populations, and it does not matter if they are in the wild or housed at a rescue center [37,38]. Some other factors as the presence or absence of avian migratory species in the area could also influence the results [38].

The presence of *Salmonella* in vultures is important for public health reasons, but also because this bacterium can play an important role in wildlife disease [39]. Outbreaks of salmonellosis in captive raptors have also been described [40]. Vultures can acquire *Salmonella* when eating contaminated livestock carcasses or in rubbish dumps. The prevalence of *Salmonella* in our study was 6.3%, which is lower than the level described by other authors for griffon vultures [22,39] and Egyptian vultures [41]. It should be pointed out that the samples used by these authors were taken at a supplementary feeding station, just after the animals consumed the carcasses of pigs, whereas our samples were taken throughout the year. Several studies have found the prevalence of *Salmonella* in raptors similar to ours [42,43,44]. Giacopello et al. [37] reported a prevalence of 9.3% in raptors admitted to a wildlife rescue center. In line with the results of Marín et al. [22], we did not find any differences in the presence of *Salmonella* between animals of different ages.

*Raoultella ornithinolytica* has been described as an emerging pathogen, associated with hospital-acquired human infections [45]. Its potential role in animal infection has yet to be studied. We consider it interesting and relevant to report the results for this species because it is an emerging pathogen, and limited information is available on its clinical features or antimicrobial susceptibility. In human beings, it has been determined that this species should never be considered a saprophytic bacteria when present in deep respiratory samples or surgical sites, and broad-spectrum treatment should be established [45]. *R. ornithinolytica* lives in an aquatic environment and can be isolated from fish [46]. Until now, it has not been described as causing animal infections, but Canarian vultures eat fish and could develop infections caused by this bacterial species, so veterinarians should be aware of this possibility.

Our results show lower percentages of isolates resistant to the antimicrobials tested than those reported by Radhouani et al. [18] for *E. coli* obtained from common buzzards, with the exception of the percentage of resistance to ampicillin, which is similar. Sharma et al. [36] found a higher percentage of resistance to ampicillin (91% vs. 51%) and a lower level of resistance to trimethoprim/sulfamethoxazole (34% vs. 50%) in *E. coli* obtained from Egyptian vultures. Their data showed a temporal variation in resistance, so it is possible that our results would be different, depending on the sampling periods.

Alcalá et al. [15] proposed that wild birds could contribute to the spreading of extended-spectrum beta-lactamase producing *E. coli*. Szmolka et al. [11] call attention to the role of antimicrobial therapy in animals in the selection of multidrug resistant populations of commensal *E. coli* that could transfer those resistance genes in vivo to pathogenic strains of *E. coli* or to *Salmonella*.

In the case of our study, vultures are sedentary, so the dissemination of antimicrobial-resistant bacteria through migration is not feasible. Therefore, the effects should be limited to the local level of the islands. To avoid these potential negative effects and others affecting the populations of this threatened subspecies, it is necessary to prevent the vultures from feeding on the remains of intensive livestock farming, particularly pigs [41], and on garbage that accumulates in rubbish dumps [47]. This study shows that Canarian vultures can be a source of antimicrobial-resistant bacteria, including those resistant to critically important antibiotics. They can spread them to other species and into the environment, and consequently may pose a risk to human and animal health.

## 5. Conclusions

The excessive use of antibiotics in the recent decades, in both human and veterinary medicine, has created what is currently one of the biggest public health problems: the rise and spread of large quantities of antibiotic-resistant bacteria. Action must be taken to avoid the set of factors that promote the increase and evolution of antibiotic resistance in wildlife, which can be easily be transferred to humans, domestic animals and the environment. However, for this to occur, a more accurate picture of the situation is needed.

The results of our study support the idea that raptors could act as a reservoir for *Salmonella* and other antimicrobial-resistant bacteria, posing a risk not only to wildlife but also to livestock and the human population. These data highlight the need to minimize the exposure of wildlife to antimicrobials through human and livestock waste and to take into account a “One Health” approach to manage the problem of antimicrobial resistance.

## Figures and Tables

**Table 1:** Global results of susceptibility tests. Antimicrobial susceptibility (%) of all isolates tested.

Antibiotic	Resistant	Intermediate	Susceptible
Ampicillin	54.25	-	45.75
Amoxicillin/Clavulanic Acid	8.5	2.85	88.65
Cephalexin	16.15	3.1	80.75
Cefpodoxime	3.1	0.77	96.13
Piperacillin	23.1	10	66.9
Imipenem	6.96	1.54	91.5
Gentamicin	11.5	-	88.5
Tobramycin	8	1.4	90.6
Amikacin	6.87	-	93.13
Enrofloxacin	17.7	3.1	79.2
Marbofloxacin	16.15	0.75	83.1
Tetracycline	48.44	1.56	50
Nitrofurantoin	6.15	10	83.85
Chloramphenicol	15.38	16.15	68.47
Polymyxin B	3.7	-	96.3
Trimethoprim/Sulfamethoxazole	46.85	-	53.15

**Table 2:** Variations in the percentages of susceptibility during the three years of the study.

Antibiotic	Resistant			Intermediate			Susceptible		
	2015	2016	2017	2015	2016	2017	2015	2016	2017
Ampicillin	53.3	50	60	-	-	-	46.7	50	40
Amoxicillin/Clavulanic Acid	10.35	25	12	-	-	8	89.65	75	80
Cephalexin	-	16.4	22.9	4.8	-	6.25	95.2	83.6	70.85
Cefpodoxime	-	4.9	2.1	-	-	2.1	100	95.1	95.8
Piperacillin	9.52	19.7	33.3	19	6.5	10.4	71.48	73.8	56.3
Imipenem	4.7	5.1	10	-	-	4	95.3	94.9	86
Gentamicin	3.33	16.4	10.4	-	-	-	96.67	83.6	89.6
Tobramycin	3.33	9.9	8.4	-	1.65	2.1	96.67	88.5	89.5
Amikacin	-	9.7	6.25	-	-	-	100	90.3	93.75
Enrofloxacin	38.1	11.47	16.67	4.76	1.64	4.16	57.14	86.89	79.17
Marbofloxacin	33.33	11.47	14.6	-	-	2.1	66.67	88.53	83.3
Tetracycline	38.1	39	64.6	-	3.4	-	61.9	57.6	35.4
Nitrofurantoin	14.3	-	10.42	-	4.9	20.83	85.7	95.1	68.75
Chloramphenicol	14.3	9.8	22.9	4.7	6.6	33.35	81	83.6	43.75
Polymyxin B	-	-	6.12	-	-	-	100	100	93.88
Trimethoprim/Sulfamethoxazole	41.9	48.4	48	-	-	-	58.1	51.6	52

**Table 3:** Susceptibility of bacteria isolated from chicks in the nests and from the rest of animals (adult and immature birds). Results of the statistical differences between chicks and the rest of the animals are presented.

Antibiotic	Age	Resistant	Intermediate	Susceptible	Chi-Squared (χ^2^)	*p*-Value
Ampicillin	chicks	46.9	-	53.1	3.404	0.065
	rest	63.9	-	36.1		
Amoxicillin/Clavulanic Acid	chicks	2.45	2.45	95.1	7.308	0.007 *
	rest	16.7	3.3	80		
Cephalexin	chicks	11.6	1.3	87.1	2.274	0.132
	rest	23.1	5.8	71.1		
Cefpodoxime	chicks	1.28	-	98.72	0.871	0.351
	rest	5.77	1.93	92.3		
Piperacillin	chicks	13.9	8.9	77.2	8.235	0.004 *
	rest	37.25	11.75	51		
Imipenem	chicks	3.8	1.3	94.9	1.942	0.163
	rest	11.8	1.9	86.3		
Gentamicin	chicks	12.8	-	87.2	0.060	0.807
	rest	9.83	-	90.17		
Tobramycin	chicks	6.4	2.6	91	0.124	0.724
	rest	9.8	-	90.2		
Amikacin	chicks	6.34	-	93.66	0.012	0.914
	rest	7.69	-	92.31		
Enrofloxacin	chicks	19.2	1.3	79.5	0.108	0.743
	rest	15.38	5.77	78.85		
Marbofloxacin	chicks	17.9	-	82.1	0.192	0.662
	rest	13.47	1.93	84.6		
Tetracycline	chicks	36.36	1.3	62.34	10.099	0.001 *
	rest	66.66	1.96	31.38		
Nitrofurantoin	chicks	5.12	6.42	88.46	0.050	0.823
	rest	7.7	15.38	76.92		
Chloramphenicol	chicks	7.7	14.1	78.2	7.448	0.006 *
	rest	27	19.2	53.8		
Polymyxin B	chicks	4.35	-	95.65	0.059	0.809
	rest	2.85	-	97.15		
Trimethoprim/ Sulfamethoxazole	chicks	45.68	-	54.32	3.404	0.065
	rest	48.38	-	51.62		

* statistical significant differences (*p* < 0.05).

**Table 4:** Percentages of antimicrobial susceptibility in different bacteria species.

Antibiotic	Species *	Resistant	Intermediate	Susceptible
Ampicillin	*E. coli*	51.3	-	48.7
	*Salmonella*	22.22	-	77.78
	Others	85	-	15
Amoxicillin/Clavulanic Acid	*E. coli*	6.2	2.8	91
	*Salmonella*	-	-	100
	Others	25	5	70
Cephalexin	*E. coli*	7.8	1	91.2
	*Salmonella*	88.89	-	11.11
	Others	26.3	15.8	57.9
Cefpodoxime	*E. coli*	3.9	-	96.1
	*Salmonella*	-	-	100
	Others	-	5.26	94.74
Piperacillin	*E. coli*	19.4	11.6	69
	*Salmonella*	22.22	11.11	66.67
	Others	44.44	-	55.56
Imipenem	*E. coli*	4.9	-	95.1
	*Salmonella*	-	11.11	88.89
	Others	21	5.3	73.7
Gentamicin	*E. coli*	5.45	-	94.55
	*Salmonella*	100	-	-
	Others	5	-	95
Tobramycin	*E. coli*	2.7	1.8	95.5
	*Salmonella*	77.78	-	22.22
	Others	5	-	95
Amikacin	*E. coli*	1	-	99
	*Salmonella*	88.89	-	11.11
	Others	-	-	100
Enrofloxacin	*E. coli*	19	2.9	78.1
	*Salmonella*	-	-	100
	Others	15.8	5.3	78.9
Marbofloxacin	*E. coli*	18.6	-	81.4
	*Salmonella*	-	-	100
	Others	10.5	5.3	84.2
Tetracycline	*E. coli*	45.5	2	52.5
	*Salmonella*	25	-	75
	Others	73.7	-	26.3
Nitrofurantoin	*E. coli*	2.95	5.88	91.17
	*Salmonella*	-	22.22	77.78
	Others	26.3	26.3	47.4
Chloramphenicol	*E. coli*	14.7	16.7	68.6
	*Salmonella*	-	-	100
	Others	26.3	21	52.7
Polymyxin B	*E. coli*	5	-	95
	*Salmonella*	-	-	100
	Others	-	-	100
Trimethoprim/Sulfamethoxazole	*E. coli*	50	-	50
	*Salmonella*	-	-	100
	Others	50	-	50

* Others: *Proteus mirabilis*, *Providencia rettgeri*, *Providencia stuartii*, *Klebsiella oxytoca*, *Klebsiella pneumoniae*, *Citrobacter braakii* and *Raoultella ornithinolytica*.
